# Characteristic patterns of microRNA expression in human bladder cancer

**DOI:** 10.3389/fgene.2012.00310

**Published:** 2013-01-04

**Authors:** Anastasia A. Zabolotneva, Alex Zhavoronkov, Andrew V. Garazha, Sergey A. Roumiantsev, Anton A. Buzdin

**Affiliations:** ^1^Laboratory of Bioinformatics, Dima RogachevFederal Research Center of Pediatric Hematology, Oncology and ImmunologyMoscow, Russia; ^2^Group for Genomic Regulation of Cell Signaling Systems, Shemyakin–Ovchinnikov Institute of Bioorganic ChemistryMoscow, Russia

**Keywords:** bladder cancer, urothelial cancer, microRNA, biomarker

## Abstract

MicroRNAs (miRNAs) are small, non-coding RNAs that post-transcriptionally regulate gene expression. Their altered expression and functional activity have been observed in many human cancers. miRNAs represent promising diagnostic and prognostic molecular biomarkers, and also serve as novel therapeutic targets. We performed a systematic analysis of scientific reports that link differences in miRNA expression with the pathogenesis of bladder cancer (BC). This literature review is the first comprehensive database of miRNA molecules with biased expression profiles in BC. Among the 95 differentially expressed miRNAs that we identified from the literature, we classify 48 as up-regulated in BC, 35 as down-regulated, and 12 as contradictory (contradictory data were reported in one or more studies on the gene). In addition, we discuss the possible roles of differentially expressed miRNAs in the regulation of intracellular signaling pathways in BC.

## MOLECULAR MARKERS OF BLADDER CANCER

Bladder cancer (BC) is one of the most common urogenital cancers. For example, approximately 386,300 new BC cases and 150,200 deaths caused by the BC worldwide were registered in 2008. The incidence of BC varies greatly among different geographic regions (ranges between 1.8 and 27.1 per 100,000 males and 0.5 and 4.1 per 100,000 females) with the highest incidences in countries where the dominant population is Caucasoid (Ploeg et al., 2009). BC accounts for 3.1 and 1.8% of the overall cancer mortality in males and females, respectively. BC has many known risk factors. As BC occurs more commonly in the elderly, age is the major risk factor. The median ages of men and women diagnosed with BC are 72 and 74 years, respectively (Jemal et al., 2011). Cigarette smoking is another major risk factor for developing BC (Brennan et al., 2000). The relative risk of death from BC among smokers is 2.75 for current smokers and 2.0 for former smokers (Jacobs et al., 2010). Many chemicals are thought to be carcinogens for BC, including aniline dyes and aromatic amines (Vineis and Pirastu, 1997). Urinary tract infection, chronic irritation from catheters or bladder stones, and a non-functioning bladder are associated with an increased risk of squamous cell carcinoma (SCC) of the bladder (Shokeir, 2004). Bladder infection by Schistosoma hematobium carries an increased risk of BC, especially SCC, and is endemic in Egypt; inflammation is thought to play an important role in carcinogenesis associated with this parasite (Mostafa et al., 1999). Exposure to pelvic radiation, for example in men with prostate cancer, appears to increase the risk of BC (Sandhu et al., 2006). Strong epidemiologic evidence does not exist for a hereditary cause of most BCs (Golijanin et al., 2006).

Urothelial carcinoma of the bladder, the most common histopathologic type of BC, has a variety of genetic and phenotypic characteristics. Many genetic factors, such as chromosomal abnormalities, genetic polymorphisms, mutations, and epigenetic alterations, contribute to tumorigenesis and the progression of this type of cancer (Knowles, 2008). The most common genetic alterations reported in these tumors are deletions on chromosome 9 and point mutations in fibroblast growth factor (FGF) receptor 3 (FGFR3) and the alpha catalytic subunit of phosphatidylinositol 3-kinase (PIK3CA). More than half of all bladder tumors (all grades and stages) contain chromosome 9 alterations suggesting that chromosome 9 genes may be involved in early tumor development.

Many of these molecular peculiarities may serve as diagnostic and/or prognostic markers of tumor growth, as well as signs of disease progression. BC diagnostics can be based on the detection of molecular markers, which can provide detailed molecular insight into the progression and metastasis of disease. The clinical use of molecular markers can therefore lead to more accurate and patient-specific prognoses and surveillance. In addition, the use of biomarkers has the potential to improve the quality of life of BC patients by limiting invasive and painful procedures traditionally used to diagnose tumor growth.

At present, the most commonly used molecular markers of BC are protein-coding genes and their products, which show differential expression in tumor cells versus normal cells. These genes include members of the *RAS* family [*FGFR3, PIC3CA, TP53, *and others (Sidransky et al., 1991; Jebar et al., 2005)], as well as differentially methylated DNA loci [for example, hypermethylated *CDH1, RASSF1A, APC, CDH13*, and other genes (Maruyama et al., 2001)]. In this review, we systematically analyzed the peer-reviewed, scientific literature that reports the identification and characterization of microRNA molecules (miRNAs) differentially expressed between cancerous bladders and normal bladders of men, and summarized the information in a database.

Small non-coding RNAs are endogenous RNA molecules of about 18–25 nucleotides in length that regulate gene expression in many ways (Kaikkonen et al., 2011). There are several classes of small RNAs like miRNAs, including PIWI interacting RNAs (piRNAs), small interfering RNAs (siRNAs), and others that are each characterized by their different targets, mechanisms of maturation, and action. In mammals, miRNAs are necessary for normal development, cell growth, differentiation, apoptosis, and the regulation of many other processes (Kusenda et al., 2006). miRNAs are also known to play significant roles in tumorigenesis. More than 50% of miRNA genes are located in cancer-associated genomic regions or in fragile sites of the genome. Many types of cancer are associated with aberrantly expressed miRNAs. Both losses and gains of miRNA function contribute to cancer development and continued tumor growth (Calin et al., 2004). miRNAs may act as both oncogenes or tumor suppressors (Shenouda and Alahari, 2009). Furthermore, different cancer types, stages, and differentiation grades may have unique miRNA expression profiles, which make miRNAs potent biomarkers for cancer diagnosis (Song and Meltzer, 2011; Corsini et al., 2012; Cortez et al., 2012; Qi and Mu, 2012).

Here, we report the first comprehensive database of BC-associated miRNAs whose expression profiles are associated with cancer. We performed a systematic search for published literature that reported the isolation and characterization of expression of BC-specific miRNAs. In the future, these differential miRNA molecules may be used for developing BC diagnostic and prognostic assays.

## BC-ASSOCIATED miRNAs

Many diagnostic and prognostic biomarkers of BC have been identified over the past decade. Most of these are the biomarkers for gene transcription, DNA methylation, or protein expression (Van Tilborg et al., 2009). Since the examination of the function of miRNAs is a relatively new discipline in the biomedical sciences, there exists a paucity of data on the prognostic role of miRNAs during BC development. Thus, it is difficult to evaluate the significance of miRNAs as indicators of tumor presence, growth, progression, recurrence, development of metastasis, or patient survival. However, pioneering data reported by several research groups enable the consideration of miRNAs as possible markers for the identification of BC patients with poor prognoses (those with the most advanced stages of disease).

Using microarrays, quantitative real-time PCR (qRT-PCR), or deep sequencing technologies, it is now possible to detect up- and down-regulated miRNAs not only in cancerous or normal tissues, but also in samples of urine, blood, or other biological fluids (Pritchard et al., 2012). Among the studies examined in the present review, the most commonly up-regulated miRNA in BC was miR-129 (Dyrskjot et al., 2009). In contrast, miR-145 and miR-133a were reported to be down-regulated in cancer tissues, and these two markers allowed the authors to distinguish cancer cells from non-cancer cells with a sensitivity >70% and specificity >75% (Ichimi et al., 2009). These down-regulated miRNA molecules are known to function as tumor suppressors by negatively regulating the expression of several oncogenes (Kent et al., 2010; Moriya et al., 2012).

Yamada et al. (2011) investigated the expression of 27 miRNAs in 104 BC tissues and in urine samples using miRNA microarray profiling and qRT-PCR. Their study demonstrated that miR-96 and miR-183 concentrations in urine were significantly correlated with tumor stage and grade. These miRNAs, therefore, can be regarded as promising diagnostic and prognostic BC markers. In addition, expression of these miRNAs significantly decreased after radical surgery, suggesting that they can also be used as prognostic molecular markers of cancer recurrence.

In another study (Wang et al., 2012), researchers analyzed miRNA concentrations in urine sediment and urine supernatant of BC and non-BC patients using qRT-PCR. The authors found that the levels of miR-200 family members – miR-141, miR-155, and miR-429 – were lower in urine sediment in BC patients. Additionally, levels of miR-200a, miR-200b, miR-200c, miR-141, and miR-429 in the urinary sediment increase significantly following surgery. In urine supernatant, the level of miR-192 was higher in the control group, whereas the level of miR-155 was higher in the cancer group. Levels of miR-200a, miR-200b, miR-200c, miR-141, miR-429, miR-205, miR-192, and miR-146a increased significantly after surgery. These findings suggest that miR-200-family miRNAs (miRs-141, -429, -192, -146a, -141, and others) are promising as non-invasive, diagnostic, and prognostic markers.

In another study (Adam et al., 2012) miRNA levels were measured in the plasma of patients with or without BC. The authors identified a total of 79 differentially expressed plasma miRNAs. Some diagnostically relevant miRNAs, such as miR-200b, were up-regulated in the BC patients, whereas others, such as miR-92 and miR-33, were inversely correlated with the clinical stage of the cancer. These findings support the notion that cell-free circulating miRNAs in the blood can be released in the bladder, as well as in many other tissues (Allegra et al., 2012). Using a panel of miRNA markers, the authors detected BC in the samples with up to 89% accuracy, and with the accuracy of 92% for distinguishing invasive BC samples from non-cancer samples. Finally, the accuracy of approximately 100% was demonstrated for distinguishing muscle-invasive BC from healthy controls, and a 79% accuracy was reported for three-way classification between muscle-invasive BC, non-muscle-invasive BC, and controls. Strong prognostic relationships were seen with upregulation of miRs-133b, -129, -518c, and with a variety of other miRNAs (Dyrskjot et al., 2009). However, further studies will be needed to validate this type of molecular diagnostics.

## miRNA BIOMARKERS FOR TREATMENT SELECTION

**FIGURE 1:**
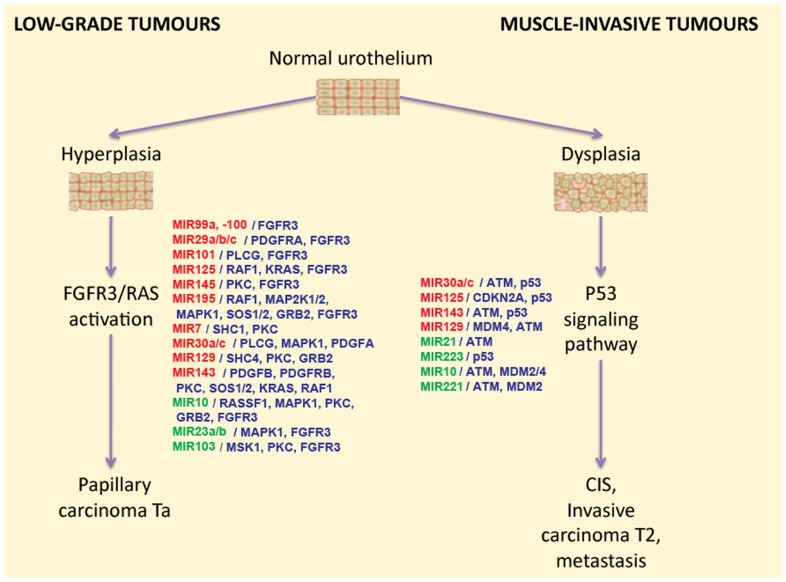
**The two-pathway model of different types of bladder cancer development, and the miRNAs that regulate these pathways**. miRNAs that were reported to be down-regulated in BC are highlighted in red, and up-regulated miRNAs are highlighted in green. miRNA target genes are shown in blue in front of the respective miRNA names.

Differentially expressed miRNAs may regulate intracellular signaling pathways. For example, it was demonstrated that this is the case for two signaling pathways associated with BC progression. Abnormal activation of the FGFR3 gene by means of either overexpression or mutation can be found in approximately 80% of non-invasive bladder tumors (Cappellen et al., 1999; Billerey et al., 2001). FGFR3 and several other growth factor receptors participate in the activation of the RAS kinase signaling pathway. Activation of this pathway leads to the increased cell proliferation, motility, and cancer transformation through hyperplasia of normal urothelium (**Figure 1**). Using bioinformatic methods, it was predicted that some miRNA molecules aberrantly expressed in BC actually target the FGFR3 gene product. Examples of this include miR-145, miR-101, miR-99a, and miR-100. Furthermore, the regulation of FGFR3 by miR-99a and miR-100 has been experimentally validated (Catto et al., 2009). Increased expression of miR-143 in BC cells is accompanied by lower expression of RAS genes. Another line of evidence recently confirmed this by showing that induced transcription of miR-143 in BC cell lines led to decreased expression of RAS (Lin et al., 2009; Kent et al., 2010). Another study demonstrates that low expression of miR-7 is associated with the hyperactive FGFR3 mutation status found in BC tissues (Veerla et al., 2009).

The second major pathway engaged in BC progression is misregulated in muscle-invasive tumors which mainly contain mutations in the TP53 gene. So far there is no proven experimental data available on the role of differentially regulated miRNA on the regulation of the p53 pathway. However, several miRNAs that are frequently found to be aberrantly expressed in BC were bioinformatically predicted to target p53 or some of its regulators (Fendler et al., 2011). For example, MDM2 [p53-binding protein homolog (mouse)], MDM4 [Mdm2-p53-binding-protein homolog (mouse)], and ataxia telangiectasia mutated (ATM) gene products were predicted as targets for miR-10. Small RNA miR-129 potentially targets the genes MDM4 and ATM in this pathway; miR-125b, miR-143, miR-30a/c, and miR-223 were predicted to target p53 directly (Fendler et al., 2011). In agreement with these findings, it was reported that expression levels of miR-10a, miR-125b, and miR-222 may serve as predictors of muscle-invasive carcinomas (Veerla et al., 2009).

Overall, these data indicate that non-invasive and muscle-invasive carcinomas display specific miRNA patterns associated with activation of different gene expression pathways. Thereby, some miRNAs may act as markers of activated signaling pathways and therefore may point researchers to the key components of the BC pathway. RNA profiling and selective targeting might, in the future, allow health professionals to develop a personalized approach to cure each tumor individually, therefore improving the efficiency of BC therapy.

## DATABASE OF miRNAs RELATED TO BC PROGRESSION

The major goal of the present review is to provide the most comprehensive list of miRNAs that are differentially expressed in BC. These molecules can potentially be used as biomarkers for BC diagnosis, prognosis, and to inform treatment strategies. For this purpose, we performed a search in the MedLine *database* for papers using the search terms *microRNA*, *miRNA*, *bladder*, *urothelial*, and *cancer*. Until October 2012, 101 such publications fitting this criterion were retrieved from the MedLine database. In spite of the obvious significance of miRNAs tumor progression, the relatively sparse data on miRNAs in bladder oncology illustrate that this area needs to be studied more intensively.

Literature review enabled us to identify a total of 95 miRNAs that are differentially expressed in BC, and seven miRNA genes that were differentially methylated in the BC versus non-cancer patients. Of the pool of a total of 95 RNAs, 35 miRNAs (34%) were reported as down-regulated, 48 (47%) as up-regulated, and 12 (12%) as “contradictory” (meaning, conflicting data were reported in one or more studies). Interestingly, miR31 located in 9p21 genomic region, was reported to be homozygously deleted in some BC cases (Veerla et al., 2009). This finding is in line with the other published data that point to the deletions of chromosome 9 as the most common type of chromosomal alterations in BC.

**FIGURE 2:**
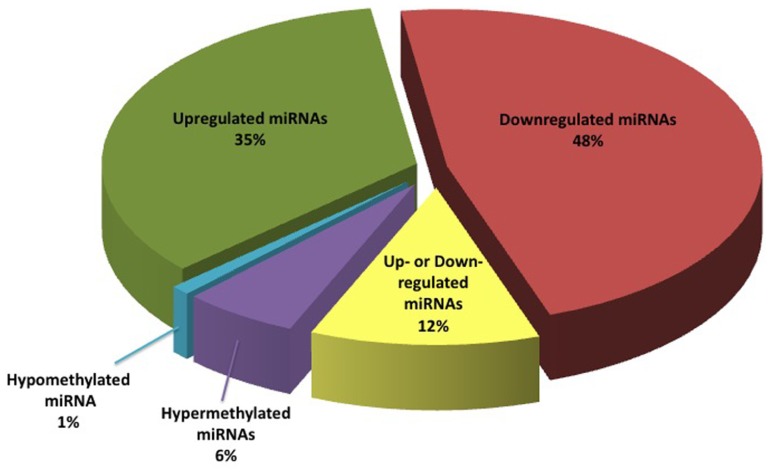
**Bladder cancer miRNA biomarker representation according to published data**.

In addition, six miRNA genes were reported to be hypermethylated in BC, while only one gene was reported as undermethylated in BC (**Figure 2**). Of these, expression data in BC is available for only one miRNA molecule (miR34). We summarized this data in a publically available database (http://bladder.pparser.net/MIRMarkers.php), which includes miRNA names, their roles in BC, and primary literature studies with supporting expression data.

This data clearly demonstrates that additional targeted, high-throughput studies are needed to investigate the relationship between miRNA expression and BC progression. The current miRNA-based two-pathway model of BC development ignores most of the reported differential miRNAs and, therefore, obviously does not reflect the complete picture of carcinogenesis in the bladder. Contemporary high-throughput methods will most likely enable finding far more participants of intracellular signaling networks regulated by the differential miRNAs involved in BC progression. Additionally, such studies would be of great utility to researchers and practitioners alike, as they may not only validate the previously published data, but also propose novel prospective biomarkers for BC diagnostics.

## Conflict of Interest Statement

The authors declare that the research was conducted in the absence of any commercial or financial relationships that could be construed as a potential conflict of interest.
